# CEP hormones at the nexus of nutrient acquisition and allocation, root development, and plant–microbe interactions

**DOI:** 10.1093/jxb/erad444

**Published:** 2023-11-08

**Authors:** Michael Taleski, Marvin Jin, Kelly Chapman, Katia Taylor, Courtney Winning, Manuel Frank, Nijat Imin, Michael A Djordjevic

**Affiliations:** Division of Plant Sciences, Research School of Biology, College of Science, The Australian National University, Canberra, ACT, 2601Australia; Division of Plant Sciences, Research School of Biology, College of Science, The Australian National University, Canberra, ACT, 2601Australia; Division of Plant Sciences, Research School of Biology, College of Science, The Australian National University, Canberra, ACT, 2601Australia; CSIRO Agriculture and Food, Canberra, ACT, 2601, Australia; Division of Plant Sciences, Research School of Biology, College of Science, The Australian National University, Canberra, ACT, 2601Australia; Department of Molecular Biology and Genetics, Aarhus University, 8000 Aarhus, Denmark; School of Science, Western Sydney University, Penrith, New South Wales 2751, Australia; Division of Plant Sciences, Research School of Biology, College of Science, The Australian National University, Canberra, ACT, 2601Australia; University of Warwick, UK

**Keywords:** Arbuscular mycorrhizal fungi, CEP peptide hormone, CEPR1, lateral root development, legume nodulation, nitrogen, root system architecture, nitrate uptake, nutrient uptake, plant–microbe interactions

## Abstract

A growing understanding is emerging of the roles of peptide hormones in local and long-distance signalling that coordinates plant growth and development as well as responses to the environment. C-TERMINALLY ENCODED PEPTIDE (CEP) signalling triggered by its interaction with CEP RECEPTOR 1 (CEPR1) is known to play roles in systemic nitrogen (N) demand signalling, legume nodulation, and root system architecture. Recent research provides further insight into how CEP signalling operates, which involves diverse downstream targets and interactions with other hormone pathways. Additionally, there is emerging evidence of CEP signalling playing roles in N allocation, root responses to carbon levels, the uptake of other soil nutrients such as phosphorus and sulfur, root responses to arbuscular mycorrhizal fungi, plant immunity, and reproductive development. These findings suggest that CEP signalling more broadly coordinates growth across the whole plant in response to diverse environmental cues. Moreover, CEP signalling and function appear to be conserved in angiosperms. We review recent advances in CEP biology with a focus on soil nutrient uptake, root system architecture and organogenesis, and roles in plant–microbe interactions. Furthermore, we address knowledge gaps and future directions in this research field.

## Teaser

This review provides an update on the roles of C-TERMINALLY ENCODED PEPTIDE (CEP) hormones with a focus on soil nutrient uptake, root system architecture, root organogenesis, and plant–microbe interactions.

## Introduction

Plants tailor their growth and development to the availability of resources to ensure survival and reproductive success. For an optimal response, plants track external resource availability (e.g. soil nutrient levels) and integrate this with the internal levels of acquired resources and demand for such resources across the entire plant body (Hermans *et al.*, 2006; Giehl and von Wirén, 2014; Wang *et al.*, 2015; Walker and Bennett, 2018). In certain cases, nutrient acquisition strategies involve symbiotic associations with soil microbes, for example legume–rhizobium nitrogen-fixing symbiosis and arbuscular mycorrhizal (AM) fungi associations with root systems for the uptake of phosphate and other nutrients (Oldroyd and Leyser, 2020). Nutrient responses involve the utilization of local and long-distance signalling molecules (Shabala *et al.*, 2016; Ko and Helariutta, 2017; Gautrat *et al.*, 2021; Wheeldon and Bennett, 2021), where there is a growing interest in the role of peptide hormones and their cognate receptors (Roy *et al.*, 2018).

C-TERMINALLY ENCODED PEPTIDEs (CEPs) are encoded by a multigene family in seed-bearing plants that responds to several stimuli including low nitrogen, high carbon, and abiotic stress (Delay *et al.*, 2013; Imin *et al.*, 2013; Tabata *et al.*, 2014; Taleski *et al.*, 2018; Chapman *et al.*, 2019). Mature 15 amino acid CEP hormones, which are derived from the post-translational modification of short pre-propeptide precursors (~80–200 amino acids), are secreted to the apoplast and can enter the xylem stream and be translocated to the shoot (Tabata *et al.*, 2014; Mohd-Radzman *et al.*, 2015; Patel *et al.*, 2018). Extracellular CEPs can bind to two CEP receptors, CEP Receptor 1 (CEPR1) and CEPR2 (Tabata *et al*., 2014), however, much less is known about the function of the interaction of CEPs with CEPR2. This review provides an update on CEP–CEPR1 function in controlling soil nutrient uptake, root system architecture, root organogenesis, and plant–microbe interactions.

## Mineral nutrition

### CEPs play a role in systemic nitrogen demand signalling

Plant roots are exposed to diverse soil environments. For example, there are heterogeneous spatial and temporal distributions of soil nutrients, as well as overall severe nutrient limitations. Therefore, plants ensure efficient nutrient uptake and appropriate root and shoot growth responses via physiological and molecular adaptations. In *Arabidopsis thaliana* (hereafter Arabidopsis) roots, these adaptations include controlling the expression and activity of nutrient transporters, and the regulation of root growth and architecture (Wang *et al.*, 2012; Jia *et al.*, 2020). For example, nitrogen (N) foraging occurs when roots are exposed to spatially heterogeneous (low and high) N levels. Here, long-distance signals initiated from both high- and low-N-exposed roots are integrated with shoot N status and demand for N to facilitate a compensatory N uptake response in parts of the root system exposed to higher N. This response correlates with preferential N transporter expression and proliferation in the root exposed to high N conditions (Ruffel *et al.*, 2011; Poitout *et al.*, 2018).

**Fig. 1.**
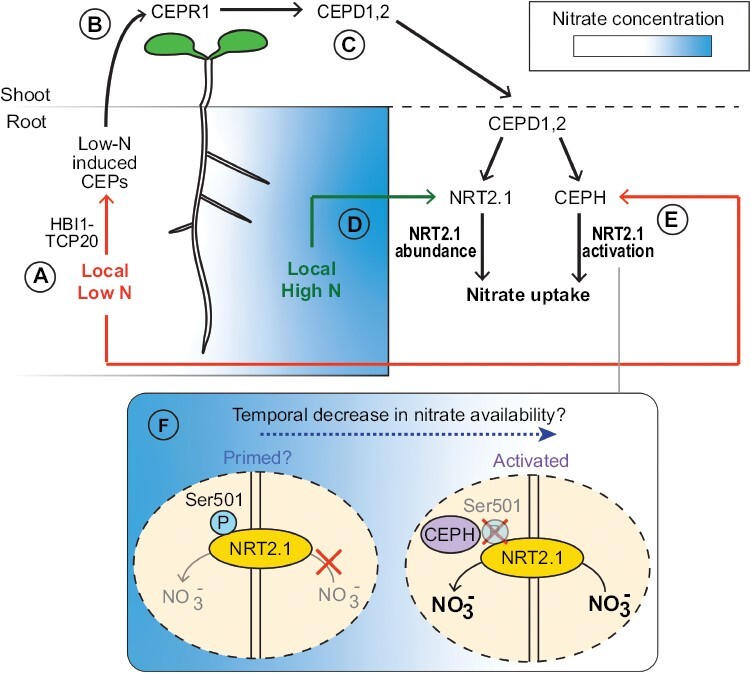
The CEP pathway coordinates systemic N demand signalling in response to heterogenous and fluctuating soil N levels. (A) *CEP* genes are up-regulated in sections of the root system exposed to low N, at least in part by the activity of the HBI1–TCP20 transcription factor module. Mature CEP peptides can translocate via the xylem stream to the shoot, where they interact with the CEPR1 receptor. (B) The interaction of CEPs with CEPR1 results in up-regulation of genes encoding CEPD1,2. (C) CEPD polypeptides translocate in the phloem to the root where they promote nitrate uptake via two responses. (D) Firstly, transcripts encoding nitrate transporter NRT2.1 are specifically up-regulated under local high N, thus promoting NRT2.1 abundance. (E) In addition, transcripts encoding the CEPH phosphatase are up-regulated. CEPH de-phosphorylates NRT2.1 at Ser501, which activates NRT2.1 transport activity (see inset). Although some *CEPH* expression occurs under high N, it is preferentially up-regulated under low N. This implies that some NRT2.1 under local high N may exist in an inactive form (i.e. phosphorylated Ser501). (F) It is possible that inactive NRT2.1 produced under high N is ‘primed’ for rapid de-phosporylation by CEPH in response to a temporal N depletion (e.g. via leaching of nitrate to lower soil strata) to activate high-affinity nitrate uptake and maintain N uptake capacity.

CEPs were shown to function as an N-demand signal (Tabata *et al.*, 2014; Ohkubo *et al.*, 2017, 2021) (Fig. 1). Tabata *et al.* (2014) showed that CEP hormones produced in roots exposed to low N enter the xylem stream and translocate to the shoot to interact with the phloem-localized receptor, CEPR1 (Fig. 1A, B). CEP binding to CEPR1 generates shoot-to-root signals that up-regulate transcripts for nitrate transporters, including high-affinity transporter NRT2.1 and dual-affinity transporter NRT1.1, in roots exposed to higher N (Tabata *et al.*, 2014). Ohkubo *et al.* (2017) identified the putative CC-type glutaredoxins, CEP DOWNSTREAM 1 (CEPD1) and CEPD2, as the phloem-mobile, shoot-to-root return signals that specifically up-regulate *NRT2.1* expression in roots exposed to localized high N (Fig. 1C, D). CEP signalling appears to play a broader role in adjusting N homeostasis outside the conditions where high-affinity nitrate transporters dominate since Arabidopsis *cepr1* knockout mutants are defective in nitrate uptake at uniform low or high N (0.2 mM and 10 mM, respectively). In addition, CEP promotion of N uptake appears conserved in *Medicago truncatula* (hereafter Medicago). Mutants defective in the CEPR1 orthologue, COMPACT ROOT ARCHITECTURE 2 (CRA2) (Huault *et al.*, 2014; Mohd-Radzman *et al.*, 2016), also have reduced root N uptake (Bourion *et al.*, 2014), and MtCEP1 peptide systemically promotes *MtNRT2.1* expression and nitrate uptake in a CRA2-dependent manner (Luo *et al.*, 2023). The induction of *CEP* genes in response to environmental stresses, such as N limitation, is also a conserved feature of CEP signalling in other plant species, including apple (*Malus×domestica*) (Li *et al.*, 2018), cucumber (*Cucumis sativus*) (Liu *et al.*, 2021), and rice (*Oryza sativa*) (Sui *et al.*, 2016).

Chu *et al.* (2021) provided insight into the mechanism by which CEP genes are up-regulated under low N in Arabidopsis. The authors showed that the physically interacting transcription factors HOMOLOG OF BRASSINOSTEROID ENHANCED EXPRESSION2 INTERACTING WITH IBH1 (HBI1) and TEOSINTE BRANCHED1/CYCLOIDEA/PROLIFERATING CELL FACTOR1-20 (TCP20) bound the promoters of multiple *CEP* members and synergistically increased their expression under low N (Fig. 1A).

### CEP signalling post-translationally activates NRT2.1

The nitrate transport activity of NRT2.1 is controlled by phosphorylation at specific residues (Jacquot *et al.*, 2020). Ohkubo *et al.* (2021) showed that CEP signalling promotes NRT2.1 post-translational activation in addition to *NRT2.1* expression (Fig. 1E). These findings have possible implications for the understanding of how plants manage to adapt to fluctuating soil N levels. First, they identified the *CEPD-INDUCED PHOSPHATASE* (*CEPH*) gene, encoding a PPC2 family phosphatase, which was induced by CEPD1,2 and another member of the CC-type glutaredoxin family, CEPD-LIKE 2. A *ceph* knockout mutant was defective specifically in high-affinity nitrate uptake. CEPH’s identity as a phosphatase suggested a role in the post-translational activation of high-affinity nitrate transport, which was confirmed by quantitative phosphoproteomics showing that CEPH de-phosphorylates Ser501 of NRT2.1. Ser501 is a phosphosite known to repress NRT2.1 activity (Jacquot *et al.*, 2020).

Together, these results suggest CEP signalling has a dual function in promoting NRT2.1 nitrate transport activity by Ser501 de-phosphorylation, in addition to promoting NRT2.1 transcript abundance (Fig. 1D, E). Interestingly, whilst CEP signalling promotes *NRT2.1* expression specifically under local high N (Ohkubo *et al.*, 2017), *CEPH* expression was maximal under low N (Ohkubo *et al.*, 2021). This implies that some of the NRT2.1 protein produced under local high N may be in an inactive (Ser501-phosphorylated) state. Ohkubo *et al.* (2021) suggested that inactive NRT2.1 produced under high N may be rapidly dephosphorylated by CEPH to activate high-affinity nitrate transport if soil N levels are low. This could represent a strategy to maintain the capacity to forage for highly mobile nutrients such as nitrate that can rapidly leach from soils (Lynch, 2013) (Fig. 1F). The authors suggest that this may be advantageous, as protein dephosphorylation is more energetically favourable than *de novo* protein biosynthesis. Further work is required to define how CEP signalling controls the function of NRT2.1 via CEPH under heterogeneous N levels.

### Roles beyond root nitrate uptake

Recently, Roy *et al*. (2022) provided evidence for a broader role for Medicago and Arabidopsis CEPs in nutrient uptake. CEP application promoted phosphate and sulfate uptake, in addition to nitrate uptake, in the high-affinity range in both species. Moreover, Kawai *et al.* (2022) suggested that CEPs may play a more specialized nutrient acquisition role in the context of ammonium uptake and assimilation in response to heterogeneous availability in the wild rice *Oryza longistaminata*. The notion that CEP signalling may also promote root-to-shoot nitrate translocation (Lin *et al.*, 2008) is supported by CEP hormone inducing *NRT1.5* up-regulation (Delay *et al.*, 2019), and *cepr1* plants showing *NRT1.5* down-regulation (Tabata *et al.*, 2014; Chapman *et al.*, 2019).

There is evidence that CEP–CEPR1 also controls N homeostasis more broadly at the whole-plant level, beyond its impacts on root N acquisition and translocation. Taleski *et al.* (2020) revealed a reproductive tissue-specific role for CEPR1 in influencing seed size and yield. Arabidopsis *cepr1* mutants had strongly decreased seed yield resulting from a suite of phenotypes including fewer ovules per silique, higher seed abortion frequency, and smaller seed size. Seed yield was primarily determined by *CEPR1* activity in the bolt tissues as demonstrated through reciprocal bolt grafting between wild-type and *cepr1* plants. *CEPR1* is expressed throughout the reproductive tissue vasculature, including in the chalazal seed coat, which is critical for nutrient delivery from the mother plant to the seed. The *cepr1* mutants displayed chlorosis and anthocyanin accumulation symptoms consistent with an impaired nitrogen status in reproductive tissues. This correlated with reduced expression in *cepr1* bolts of key nitrogen assimilation (*GLUTAMINE SYNTHETASE 1;2*) and transport (*USUALLY MULTIPLE ACIDS MOVE IN AND OUT TRANSPORTERS 14*) genes known to be involved in nitrogen remobilization and delivery to seeds (Müller *et al.*, 2015; Moison *et al.*, 2018). Altered expression of several *CEP* genes in *cepr1* bolts, which is indicative of feedback/feedforward regulation, implies a reproductive tissue-localized CEP–CEPR1 circuit affecting seed size and yield, possibly by affecting nitrogen remobilization for seed filling. Findings from several other species also support a role for CEP–CEPR1 signalling in reproductive development, including in tomato (*Solanum lycopersicum*) (Takei *et al.*, 2019), maize (*Zea mays*) (Xu *et al.*, 2021), and rice (Ogilvie *et al.*, 2014; Sui *et al.*, 2016). These findings are particularly interesting, given that the evolution of CEP genes and CEPR1 correlates with the emergence of seed-bearing plants (Ogilvie *et al.*, 2014; Furumizu *et al.*, 2021; Furumizu and Aalen, 2023).

### Unresolved questions

It is not known if CEP signalling plays a role in the differential root growth response seen under heterogeneous N conditions. A role for CEPs in promoting the root proliferation response in local high-N patches has not been ruled out experimentally; however, it is unlikely given that CEPs act via the shoot as a systemic inhibitor of root growth in Arabidopsis (Taleski *et al.*, 2023). Although CEPDs are putative glutaredoxins, it is not known if they act to regulate redox or how they regulate gene expression from the vascular tissue to the tissues external to the stele where nutrient transporters function. However, there is cellular evidence that glutaredoxins can translocate from the stele to outer tissue layers (Ohkubo *et al.*, 2017; Ota *et al.*, 2020), potentially through plasmodesmata. It is not known how CEP signalling controls the uptake of other nutrients beyond nitrate (Roy *et al.*, 2022). An emerging theme is that CEP-mediated signalling may coordinate a broader range of nutrient uptake pathways so that growth is metered to match whichever nutrient most limits growth.

## Root system architecture

### Arabidopsis CEPs inhibit primary root growth in response to nutrient limitation

Primary root growth is a major determinant of the overall root system depth, which affects access to water and nutrients in lower soil strata (Lynch, 2013). Primary root growth contributes to the establishment of young seedlings before lateral root (LR) emergence (Hanslin *et al.*, 2019; Taylor *et al.*, 2021). In contrast to nutrient foraging responses that promote root growth under mild nutrient limitation, severe nutrient limitation results in a survival strategy involving the inhibition of primary root growth (Gruber *et al.*, 2013; Li *et al.*, 2017; Weiste *et al.*, 2017). This cessation of root tip growth under nutrient starvation is typified by an exit of meristematic cells from the cell cycle into a state of mitotic quiescence, which is reversible upon nutrient resupply and correlates with the activity of the energy sensor TARGET OF RAPAMYCIN (TOR) (Li *et al.*, 2017). One of the best-established responses to CEP hormone addition or overexpression in Arabidopsis is the inhibition of primary root growth (Ohyama *et al.*, 2008; Delay *et al.*, 2013; Roberts *et al.*, 2016). Delay *et al.* (2013) showed that *CEP3*, which is up-regulated under N starvation and other conditions such as salinity and osmotic stresses, has a role in the inhibition of primary root growth in Arabidopsis. Here, a *CEP3* knockout mutant displayed increased primary root growth under a range of stress conditions including N starvation (Delay *et al.*, 2013).

Delay *et al*. (2019) used assays measuring cell cycle activity to demonstrate that CEP3 promoted the entry of primary root meristems into mitotic quiescence under carbon (C), N, or a combined C and N starvation. Given that CEP signalling is also known to promote uptake of nitrate and other nutrients such as phosphorus (P) and sulfur (S) (Tabata *et al.*, 2014; Roy *et al.*, 2022), it is possible CEPs function under severe nutrient limitation to simultaneously pause root growth and scavenge soil nutrients using high-affinity transporters. CEP3 also inhibited cell cycle re-entry in the primary root meristem upon nutrient resupply, independently of TOR activity (Delay *et al.*, 2019). Given that several CEP genes are up-regulated in response to high C levels (Chapman *et al.*, 2019), CEPs may possibly function as a ‘brake’ signal under nutrient imbalance to prevent premature re-establishment of root growth by C provision until limitations in other nutrients are ameliorated.

Delay *et al*. (2019) showed that CEP3 inhibits primary root meristem cell number in a CEPR1-dependent manner. Both root and shoot CEPR1 activity appeared to contribute to this; however, primary root growth inhibition by CEP3 predominantly occurs systemically via the shoot (Taleski *et al.*, 2023). This is mediated in part by shoot-to-root mobile CEPD glutaredoxins (Taleski *et al.*, 2023), which were previously characterized in CEP-dependent N acquisition pathways (Ohkubo *et al.*, 2017). This long-distance CEP signalling is consistent with the notion that growth responses in the primary root tip are integrated with whole-plant nutrient status (Xiong *et al.*, 2013; Chen *et al.*, 2016; Weiste *et al.*, 2017).

### CEP and cytokinin signalling intersect to inhibit Arabidopsis primary root growth

**Fig. 2.**
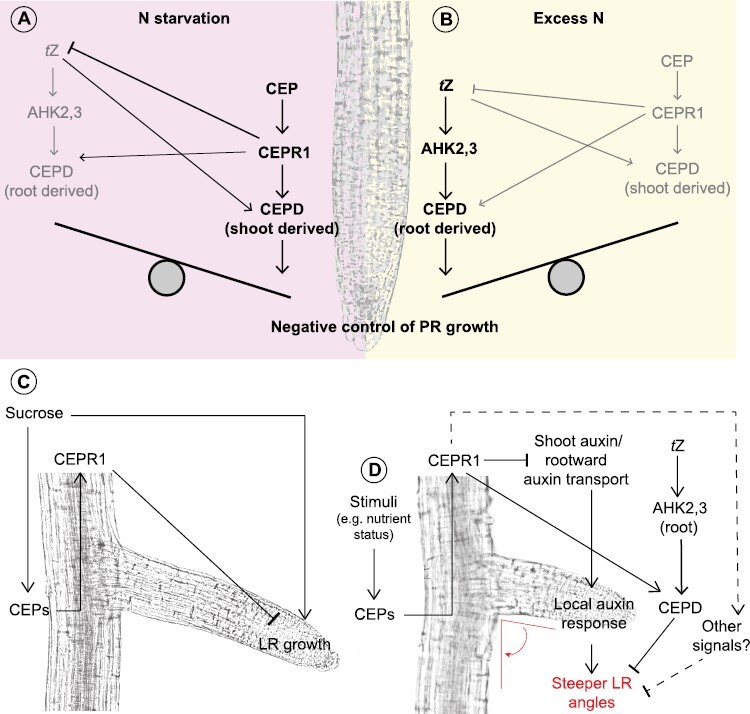
CEP signalling inhibits primary root growth, supresses the sucrose enhancement of lateral root (LR) growth, and promotes a shallower root system. (A and B) A proposed model for the interplay between CEP and cytokinin signalling in the inhibition of primary root growth under N starvation or excess. (A) Under N starvation conditions where CEP hormone levels are elevated, CEPD-dependent root inhibition occurs predominantly via shoot-derived CEPD polypeptides produced in response to CEP signalling via CEPR1. CEP signalling curtails *t*Z cytokinin production in the root, although some level of root-to-shoot *t*Z transport also appears necessary for maximal shoot *CEPD* up-regulation under low N. (B) Under excess N, where *t*Z hormone levels are elevated, CEPD-dependent primary root inhibition occurs predominantly via root-derived CEPD polypeptides produced in response to *t*Z signalling via AHK2,3 cytokinin receptors. In addition, local CEP signalling probably contributes to the pool of root-derived CEPD. Thus, CEP and cytokinin hormones converge on CEPDs to fine-tune primary root growth responses to N levels. (C) CEP signalling dampens the promotion of LR growth by sucrose. Sucrose, in addition to driving LR growth, up-regulates the expression of several *CEP* genes, resulting in the production of CEP hormones. The CEP hormones signal through CEPR1 in the root and shoot to inhibit LR growth, thus curtailing sucrose enhancement of LR growth. (D) CEP signalling promotes a shallower root system in response to environmental cues. Stimuli such as low N or elevated C result in increased CEP production. CEPs interact with CEPR1 in the shoot, which results in decreased rootward auxin, and a dampened local auxin response in LRs. Thus, local auxin responses that promote LR orientation towards the gravity vector (i.e. steeper LR angles) are inhibited by CEP signalling, resulting in a shallower root system. Additionally, CEP signalling via CEPR1 and *t*Z signalling via AHK2,3 converge on CEPD activity to promote shallower LR angles. It is possible that other long-distance signals downstream of CEPR1 also contributes to the control of LR angles.

Recent advances suggest that CEP and cytokinin pathways intersect with each other (Fig. 2A, B). Like CEPs, cytokinins regulate plant growth responses to the environment and nutritional status (Werner and Schmülling, 2009; Kieber and Schaller, 2014; Cortleven *et al.*, 2019). CEP and cytokinin mutants have similar phenotypes, which hinted at a potential interaction between the pathways. Double-knockout mutants in ARABIDOPSIS HISTIDINE KINASE 2 (AHK2) and AHK3 cytokinin receptors, as well as *cepr1* knockout mutants, both have increased root growth and stunted shoot growth (Riefler *et al.*, 2006; Chang *et al.*, 2013; Tabata *et al.*, 2014; Chapman *et al.*, 2019). Moreover, CEP3 and cytokinin signalling inhibits root apical meristem cell number (Dello Ioio *et al.*, 2007; Delay *et al.*, 2019), promotes a shallower angle of LR growth trajectory (Waidmann *et al.*, 2019; Chapman *et al.*, 2020), inhibits auxin transport (Růžička *et al.*, 2009; Chapman *et al.*, 2020), and promotes seed yield (Bartrina *et al.*, 2011; Taleski *et al.*, 2020). In legumes such as Medicago and *Lotus japonicus* (Lotus), CEPs and cytokinin promote nodule organogenesis (Lin *et al.*, 2020).

How CEP and cytokinin pathways intersect to regulate development was elusive until recently. A first connection between both hormones was established by showing that *CEPD1* and *CEPD2* up-regulation upon low N requires cytokinin transport through ATP-BINDING CASSETTE G14 (ABCG14) (Ota *et al.*, 2020). Since then, CEPDs were identified as a convergence point for CEP and cytokinin signalling. Both hormones contribute to a CEPD pool in the root, which is required to inhibit root growth (Taleski *et al.*, 2023). On the one hand, root-derived CEP3 contributes to this pool systemically by up-regulating CEPD expression through CEPR1 in the shoot (Fig. 2A). CEPDs are then transported to the root via the phloem stream. On the other hand, cytokinin induces *CEPD* expression locally in the root through AHK2 and AHK3 (Fig. 2B). The importance of CEPDs for CEP and cytokinin signalling is underscored by the fact that a *cepd1,2* mutant was partially insensitive to both hormones (Taleski *et al.*, 2023). In addition, CEP signalling appeared to be involved in the feedback inhibition of root cytokinin biosynthesis, with *cepr1* mutants displaying increased levels of *trans*-zeatin- (*t*Z) type cytokinins in roots. The intersection of CEP and cytokinin signalling probably allows the plant to fine-tune root growth under a range of environmental stimuli (Fig. 2A, B). For example, whilst numerous CEPs are up-regulated in response to N starvation, cytokinin levels are elevated under excess N (Takei *et al.*, 2004), where they have been previously characterized to inhibit primary root growth via the activity of glutaredoxin genes closely related to CEPDs (Patterson *et al.*, 2016). Interestingly, both hormones are responsive to elevated C levels (Chapman *et al.*, 2019; Kiba *et al.*, 2019), so combinatorial effects of C and N are likely to be important in CEP and cytokinin pathway interactions.

### Unresolved questions

Whilst there is a clear role for Arabidopsis CEP3 in inhibiting primary root growth under N starvation, roles under other stresses (e.g. salinity, low light stress, or osmotic stress) require further characterization. In addition, specific roles for other CEP genes more broadly in primary growth inhibition remain obscure. Knockdown of *CEP5* (Roberts *et al.*, 2016), or single CRISPR/Cas9 [clustered regularly interspaced palindromic repeats (CRISPR)/CRISPR-associated protein 9] knockouts of *CEP 1–8*,*12–15* genes (Huang *et al.*, 2023), resulted in increased primary root growth, notably under sufficient N, which suggests that other CEP genes contribute to controlling plant growth under conditions other than N limitation. Moreover, how CEPD specifically affects root tip growth is unknown, and other signals downstream of CEP perception by CEPR1 that contribute to primary root growth inhibition are also yet to be determined. Defining specifically how CEP and cytokinin signalling coordinates root growth in response to different environmental stimuli, such as nutrient levels or abiotic stresses, requires further work.

Much of the work investigating CEP inhibition of primary root growth has been carried out in Arabidopsis. Some work utilizing peptide addition has shown that CEP inhibition of root growth appears to be conserved in rice (Sui *et al.*, 2016) and maize (Xu *et al.*, 2021); however, these studies lacked CEP or CEPR1 gene loss-of-function mutant analyses. Cross-activity of CEP peptides is also demonstrated by the fact that CEP peptides from different species can substitute for each other to regulate root growth. For example, CEP peptides from maize can inhibit primary root growth in Arabidopsis (Xu *et al.*, 2021), and Medicago CEP1 can inhibit root growth in Arabidopsis and bind to Arabidopsis shoot vasculature in an AtCEPR1-dependent fashion (Lee *et al.*, 2021). Together, these findings demonstrate that CEP signalling probably has at least some degree of functional conservation across species. However, the full extent to which CEP signalling via CEPR1 inhibits primary root growth, particularly in species of agricultural significance, remains to be determined.

### CEP signalling negatively regulates lateral root density and elongation

Although primary roots derived from seed embryonic tissues are critical for seedling establishment, LRs form most of the mature root system. LRs provide anchorage and allow plants to explore the soil to acquire water and nutrients. Therefore, the control of LR density and length is essential for plants to balance nutrient acquisition with resource expenditure. Given its importance, many pathways, including CEP signalling, control LR growth and density (Fukaki and Tasaka, 2009; Jeon *et al.*, 2021).

Prior work showed that CEP signalling negatively regulates LR number in Arabidopsis and Medicago (Delay *et al.*, 2013; Imin *et al.*, 2013; Mohd-Radzman *et al.*, 2015, 2016; Roberts *et al.*, 2016; Taleski *et al.*, 2016). Chapman *et al.* (2019) showed that the CEP–CEPR1 pathway in Arabidopsis decreased LR growth by reducing LR meristem size and the length of mature LR cells. CEP signalling also attenuated the sucrose- and photosynthesis-dependent increases in LR meristem size and length, probably through a sucrose-dependent up-regulation of a subset of the CEP multigene family (Fig. 2C). RNA-seq analyses showed that many of the genes with basally altered transcription in *cepr1* corresponded to Sucrose non-Fermenting Related Kinase 1 (SnRK1)-dependent targets (Baena-González *et al.*, 2007), which suggested that C signalling is perturbed in *cepr1* roots (Chapman *et al.*, 2019). Therefore, CEP signalling acts to control LR proliferation not only in response to N limitation but also in response to C availability or possibly an imbalance in C to N levels.

Recently Huang *et al*. (2023) generated a collection of Arabidopsis CEP knockout lines (CEP 1–8,12–15) using CRISPR/Cas9 to explore the role of individual CEP genes in LR growth. They found that all the CEP knockout lines consistently showed an increase in LR number and density, in accordance with earlier studies (Delay *et al.*, 2013). This indicates that CEP genes may act cooperatively to repress LR density. In addition, CEP4 and CEP8 knockout lines had increased LR length, a phenotype not observed in other individual CEP knockout lines (Huang *et al.*, 2023). To study the effect of CEP signalling in Medicago, Zhu *et al.* (2021) grew plants on media containing synthetic CEP peptides (MtCEP1, 2, 4–6, 8, and 12), which caused a significant reduction in LR number. Consistent with previous studies (Huault *et al.*, 2014; Mohd-Radzman *et al.*, 2016), Zhu *et al.* (2021) confirmed an increased LR density in *cra2*, and the lack of response of *cra2* to CEP treatment, Together, these results suggest partially redundant roles for CEPs in controlling LR number.

There is some work hinting at a role for CEPs in the development of specialized LR organs present in some species including *Lupinus albus* (white lupin) called cluster roots, which are an adaptation for P acquisition in P-poor soils. Zhou *et al.* (2019) found that the progression of cluster root development was inversely correlated to *LaCEP1* expression, and LaCEP1 peptide addition or gene overexpression inhibited cluster root development. More work is required to genetically dissect if and how CEP signalling is involved in cluster root formation and function.

Taken together, these studies indicate that CEP signalling acts in several species to negatively control LR development, and *CEP* genes appear to be up-regulated or down-regulated in different contexts to facilitate root responses to nutrient limitation or imbalances.

### CEP signalling influences the growth trajectory angle of lateral roots

The angle at which shoots and roots emerge from the plant body with respect to the gravity vector is called the gravitropic setpoint angle (GSA). The GSA is a critical determinant of overall root system shape, and it has important applications in agriculture (Roychoudhry *et al.*, 2013). Steeper angled root systems are often, but not always, seen as a physiological response to low N and, in Arabidopsis, low P conditions can also induce steeper LR GSAs (Roychoudhry *et al.*, 2017).

Although several metabolite hormones play a role in setting the GSA of LRs (Rosquete *et al.*, 2013; Roychoudhry *et al.*, 2013; Waidmann *et al.*, 2019), important roles for CEP signalling in GSA were discovered recently (Chapman *et al.*, 2020, 2024) (Fig. 2D). Chapman *et al.* (2020) demonstrated that both Arabidopsis *cepr1* and Medicago *cra2* lines have a 10–20° steeper LR GSA than their respective parental lines, and a compact and denser root system (Chapman *et al.*, 2020). Concordantly, the application of CEPs to Medicago or Arabidopsis plants causes LRs to grow at a 7–15° shallower angle. CEP promotion of shallower roots requires perception via CEPR1. Grafting experiments demonstrated that CEP–CEPR1 signalling controls LR GSA via shoot to root systemic signalling in both Arabidopsis and Medicago.

CEPs interact with multiple hormone pathways to affect GSA. For example, CEP and auxin pathways interact to control LR GSA (Fig. 2D) (Chapman *et al.*, 2020). Steeper LR GSA in *cepr1* and *cra2* correlated with increased shoot-to-root auxin transport, and the CEP receptor mutant LR GSA could be restored to wild-type levels by applying auxin transport inhibitors. In addition, *cra2* mutants demonstrated elevated shoot auxin, suggesting that CEP–CRA2 normally inhibits auxin biosynthesis in the shoot. This systemic effect of CEP–CRA2 on LR GSA via a repression of shoot auxin levels and/or shoot-to-root transport (Chapman *et al.*, 2020) contrasts the local role of CEP–CRA2 in the inhibition of LR number via a reduction in auxin synthesis in roots (Zhu *et al.*, 2020). Cytokinins, like CEPs, promote a shallower LR GSA by offsetting the positive gravitropism elicited by auxin (Waidmann *et al.*, 2019). Recently, Chapman *et al.* (2024) showed, using agar- and soil-based assays, that the CEPR1 receptor is required for the cytokinin *t*Z-mediated promotion of shallow LR angles. This signalling occurs via the cytokinin receptors AHK2 and 3, through the root. Chapman *et al.* (2024) also showed that CEP and cytokinin signals converge on CEPD1 and CEPD2 to partially regulate LR angles.

### Unresolved questions

Given that CEP and cytokinin interact to control root growth, and that cytokinin, like CEP, inhibits auxin transport (Růžička *et al.*, 2009), it will be interesting to determine if there is an interplay of these three hormones in controlling LR GSA. In addition, the mechanism of how CEPR1 signalling from the shoot affects LR gravitropism via mobile signals is not known. Whilst shoot-to-root auxin transport and CEPDs appears to be involved, it is possible other mobile signals such as miR2111 also contribute (Fig. 2D). Intriguingly, miR2111, which is a confirmed shoot-to-root signal downstream of CRA2 in Medicago (Gautrat *et al.*, 2020; see below), was identified as responsive to low phosphate in Arabidopsis (Hsieh *et al.*, 2009), but it is unclear how miR2111 affects root development. Finally, the impact of CEP control of LR GSA on nutrient acquisition needs to be determined. Curiously, although CEP signalling enhances nitrate and phosphate uptake, it promotes a shallower root system, which is thought to be more optimal for acquisition of phosphate rather than nitrate (Lynch, 2019). Alternatively, as nitrate uptake enhances low phosphate responses (Medici *et al.*, 2019), it is possible that CEP promotion of a shallow root system is a phosphate acquisition strategy, whereby potential trade-offs in nitrate acquisition are minimized through simultaneous up-regulation of nitrate transporter activity by CEP.

## Symbiosis

### CEP signalling promotes legume nodulation for symbiotic N fixation under low soil N

Under low N, certain legume species form an endosymbiotic relationship with soil bacteria, generically called rhizobia, that carry out N fixation in specialized root organs called nodules (Roy *et al.*, 2020). Rhizobia fix atmospheric N_2_ into bioavailable nitrogenous compounds for the plant in exchange for carbohydrates as sustenance. Like LR growth and high-affinity nutrient uptake, symbiotic N fixation is energetically costly and must be carefully regulated by the plant and balanced against the cost of other N acquisition strategies such as uptake of nutrients by LRs.

**Fig. 3.**
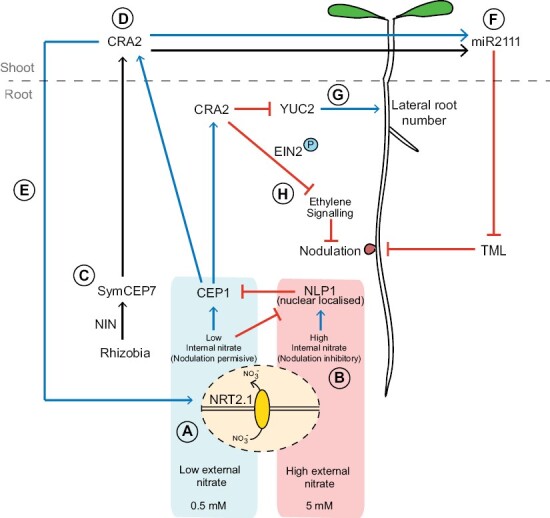
CEP signalling in legumes promotes nodulation and nitrate uptake, and differentially impinges on LR development. (A) Under low nitrate conditions, NRT2.1 facilitates the uptake of low, permissive levels of nitrate, which promote CEP1 production and nodulation. (B) Under high nitrate conditions, however, NRT2.1 activity results in accumulation of internal nitrate to levels that trigger translocation of NLP1 from the cytosol to the nucleus. Here, NLP1 directly represses *CEP1* gene expression, thus inhibiting nodulation. (C) Separately, perception of rhizobial Nod factors results in up-regulation of the NIN transcription factor, which directly promotes *CEP7* gene expression and thus production of the SymCEP7 peptide. (D) Both CEP1 and SymCEP7 peptides translocate in the xylem stream from the root to the shoot, where they can interact with the CRA2 receptor for long-distance signalling. (E) In one pathway branch, CEP1 interactions with shoot CRA2 result in return shoot-to-root signals that up-regulate *NRT2.1* expression and promote nitrate uptake from the soil. (F) In addition, both CEP1 and SymCEP7 interactions with shoot CRA2 promote nodulation by inducing the production of miR2111, which travels to the root where it decreases the abundance of transcripts for the nodulation-inhibitory TML1/2. (G) In a local circuit involving root CRA2, CEP1 but not SymCEP7 inhibits LR number by repressing YUC2 expression and thus inhibiting auxin biosynthesis. (H) CEP1 is also involved in local signalling with root CRA2 to promote nodulation. Here, CEP–CRA2 interactions result in phosphorylation of MtEIN2, which prevents cleavage of the MtEIN2 C-terminal signalling domain and thus dampens nodulation-inhibitory ethylene signalling.

Work in legumes showed that CEP signalling reduces LR number and promotes nodule number, size, and effectiveness (Imin *et al.*, 2013; Mohd-Radzman *et al.*, 2016) (Fig. 3). In the model legume, Medicago, low soil N availability induces *CEP1* transcription and the production of CEP1 hormones in the roots (Imin *et al.*, 2013; Djordjevic *et al.*, 2015; Mohd-Radzman *et al.*, 2015; Patel *et al.*, 2018). CEP1 application also counteracts the suppressive effect of high nitrate availability on nodule number and development (Imin *et al.*, 2013). Genetic and grafting studies in Medicago, and proteomic analyses of soybean (*Glycine max*) xylem sap (Okamoto *et al.*, 2015; Patel *et al.*, 2018), collectively suggest that CEPs translocate in the xylem stream to the shoot where they probably interact with a CEPR1 orthologue to positively control root nodulation (Huault *et al.*, 2014; Mohd-Radzman *et al.*, 2016).

### *CEP1* expression is inhibited by a nitrate-sensing transcription factor

Recent Medicago research has identified the transcription factor NODULE INCEPTION LIKE PROTEIN 1 (NLP1) and the nitrate transporter NRT2.1 as key components of a nitrate sensing mechanism that tailors *CEP1* expression to soil nitrate levels (Fig. 3A, B) (Luo *et al.*, 2022). The authors showed that NRT2.1 facilitated the uptake of low, permissive amounts of nitrate required for maximal *CEP1* induction at low, but not zero, external nitrate (i.e. 0.5 mM KNO_3_) (Fig. 3A). At high external nitrate (e.g. 5 mM), however, NRT2.1 nitrate uptake activity facilitates internal nitrate accumulation to levels that promote the migration of NLP1 from the cytosol to the nucleus. Here, NLP1 binds to a repressor element present in the *CEP1* promoter to inhibit *CEP1* expression (Fig. 3B). Thus, NRT2.1 activity appears to provide information on external nitrate availability, which is sensed internally by NLP1. Nuclear-localized NLP1 subsequently curtails the level of CEP1 signalling in accordance with soil nitrate levels, presumably to prevent unnecessary expenditure on nodulation where soil nitrate uptake by LRs is sufficient to meet N demand. This study offers the first insight into the mechanism that controls MtCEP1 transcription in response to nitrate availability.

### CEP1 signalling promotes nodulation via accumulation of shoot-to-root mobile miR2111

Gautrat *et al*. (2020) showed that the perception of CEP1 by CRA2 in the shoot increases the production of a mobile shoot-to-root miRNA, miR2111, which directly targets and reduces the accumulation in roots of transcripts encoding the Kelch repeat-containing F-box proteins TOO MUCH LOVE1 (TML1) and TML2 (Tsikou *et al.* 2018) (Fig. 3A, D, F). TML1 and TML2 are negative regulators of nodulation, and thus miR2111 increases the competence of the root for nodulation (Tsikou *et al.*, 2018). TML1,2 are components of the Autoregulation of Nodulation pathway (AON), a systemic negative feedback which limits nodule number once the first nodule organogenesis events have been initiated (Reid *et al.*, 2011; Gautrat *et al.*, 2019; Lin *et al.*, 2020). Therefore, under N deprivation, the AON pathway is inhibited by the CEP1–CRA2 dependent up-regulation of miR2111, which promotes N acquisition via symbiotic N fixation in root nodules.

### Specific MtCEP ligands differentially impinge on the development of nodule and lateral root organogenesis

Since the production of root organs is energetically costly, the level of investment in LRs versus nodules needs to be tightly regulated (Lohar *et al.*, 2004; Gonzalez-Rizzo *et al.*, 2006; Ding and Oldroyd, 2009). In contrast to its role in promoting nodulation via miR2111, CEP1 acts through CRA2 locally in roots (Huault *et al.*, 2014; Mohd-Radzman *et al.*, 2016) to reduce expression of the key auxin biosynthesis gene *Medicago YUCCA2* (*MtYUC2*) (Zhu *et al.*, 2020), thus reducing root auxin accumulation and preventing LR formation (Fig. 3A, G).

Despite the costs of additional organ growth, nodulating plants need to maintain LR growth to facilitate the uptake of water and other essential mineral nutrients. Recently, Ivanovici *et al.* (2023) used MS to determine the structure of a variant derived from the CEP7 gene (designated SymCEP7) that promotes nodulation without compromising LR growth (Fig. 3C, D, F). *CEP7* is distinguished from other MtCEP family members in that its expression is rapidly and specifically up-regulated by the common symbiosis (SYM) signalling pathway in the nodulation zone upon rhizobial infection or synthetic Nod factor treatment in a NODULE INCEPTION (NIN)-dependent manner (Jardinaud *et al.*, 2016; Laffont *et al.*, 2020; Ivanovici *et al.*, 2023) (Fig. 3C). The nodule-derived peptide counteracts the effects of AON by up-regulating shoot miR2111 expression (Ivanovici *et al.*, 2023) (Fig. 3F). In contrast to CEP1 and other variants of CEP7, SymCEP7 effects on nodulation are decoupled from LR inhibition, thus enabling nodulation without further reducing LR formation (Ivanovici *et al.*, 2023). Notably, SymCEP7 was able to increase root nodule number via application to shoots in the subnanomolar to nanomolar range. The SymCEP7 pathway may thus permit some LR growth in nodulating plants to facilitate acquisition of water and other nutrients from the soil, or to allow the plant to eventually pivot away from a nodule-focused strategy for N acquisition.

### A local role for CEP signalling in promoting nodulation by dampening ethylene signalling

CEP hormones also appear to act locally to promote nodulation by inhibiting ethylene signal transduction in the root (Mohd-Radzman *et al.*, 2016; Zhu *et al.*, 2020) (Fig. 3A, H). Ethylene produced during rhizobial infection reduces nodulation competence locally (Oldroyd *et al.*, 2001; Varma Penmetsa *et al.*, 2008). Recently, Zhu *et al.* (2020) proposed a mechanism for CEP inhibition of ethylene signalling. The authors showed that CEP1-dependent autophosphorylation of CRA2 allows the direct phosphorylation of the ethylene response pathway component Medicago ETHYLENE-INSENSITIVE2 (MtEIN2) (Fig. 3H). The phosphorylation of C-terminal residues Ser643 and Ser924 prevents the cleavage of the MtEIN2 protein, and the C-terminal EIN2 domain remains attached to the endoplasmic reticulum (ER) and, therefore, it is unable to translocate to the nucleus for promote ethylene-dependent transcription. Interestingly, this study also identified populations of CRA2 on both the plasma membrane (PM) and the ER, which would enable a direct interaction between ER-localized MtEIN2 and a potentially ER-localized CRA2. It is not known if CRA2 is able to interact with CEPs at the ER membrane. Nevertheless, these results support the CEP1–CRA2 pathway promoting nodulation by both systemic and local mechanisms.

### CEP and CLE hormones act antagonistically to fine-tune nodulation

In addition to interactions with pathways of metabolite hormones such as ethylene, CEPs also interact with CLAVATA (CLV)/EMBRYO SURROUNDING REGION (ESR)-RELATED PROTEIN (CLE) peptide hormone signalling to control nodulation. In legumes, a specific subset of CLE hormones act to inhibit nodulation via interactions with orthologues of the Arabidopsis CLV1 receptor (Hazak and Hardtke, 2016), such as HYPERNODULATION ABERRANT ROOT1 (HAR1) and SUPER NUMERIC NODULES (SUNN) in Lotus and Medicago, respectively (Carroll *et al.*, 1985; Wopereis *et al.*, 2000; Krusell *et al.*, 2002; Nishimura *et al.*, 2002; Searle *et al.*, 2003; Schnabel *et al.*, 2005; Ferguson *et al.*, 2014). In Medicago AON, root-derived CLE12 and CLE13 peptides activate shoot SUNN, causing a down-regulation of the shoot-to-root mobile miR2111, thus counterbalancing the actions of CEP signalling (Laffont *et al.*, 2019, 2020; Gautrat *et al.*, 2020). The addition of active CLE13 peptides to shoots can completely shut down nodulation in the roots (Imin *et al.*, 2018). Intriguingly, both CLE/SUNN and CEP/CRA2 pathways are under the control of cytokinin and NIN (Laffont *et al.*, 2020). The antagonistic nature of CLEs and CEPs is further supported in the context of N-induced inhibition of nodulation. Here, NLP1 acts bivalently by activating the expression of *CLE35*, a negative regulator of nodulation, and by repressing the expression of the positive regulator *CEP1* (Luo *et al.*, 2021, 2022; Moreau *et al.*, 2021). Therefore, multiple components controlling nodulation appear to intersect in an opposing fashion on the CEP and CLE pathways, which enables a dynamic fine-tuning of nodule number.

### Unresolved questions

One unanswered question is why SymCEP7 only affects nodule number and not LR number. Clearly the hydroxylation pattern of CEP7 and the amino acid composition at position 9 affect its activity, but the basis for how SymCEP7 interactions with CRA2 only affect nodulation is not known. One possibility, given that LR number is determined locally in the root by CRA2, is that SymCEP7 may preferentially bind to shoot CRA2. One hypothesis for how this could occur is that there are organ-dependent differences in CRA2–co-receptor combinations that affect receptor complex affinity for SymCEP7. Future studies should aim to define putative co-receptors that act with CRA2/CEPR1 to bind CEP peptides.

The mechanism by which a secreted CEP peptide activates CRA2 for phosphorylation of ER-localized EIN2 is not yet known, though it could involve endocytosis of activated CRA2 at the PM akin to other related plant receptors (Russinova *et al.*, 2004; Geldner *et al.*, 2007), or potentially cross-membrane phosphorylation events via plant ER–PM contact sites (Haj *et al.*, 2012; Wang *et al.*, 2017). Nevertheless, further study is needed to define the precise subcellular dynamics of CEP–CRA2–EIN2 interactions.

Our current knowledge on how CEPs regulate nodulation is largely limited to Medicago, although phylogenetic evidence exists for a legume-specific clade (Ivanovici *et al.*, 2023) and CEP addition promotes nodulation in other legume species (Imin *et al.*, 2013; Ivanovici *et al.*, 2023). A *GmCEP6* loss-of-function study points to a conservation of CEP function as a positive regulator of nodulation in soybean (Wang *et al.*, 2023, Preprint). Expression analyses of *CEP* genes in pea (*Pisum sativum*) (Lebedeva *et al.*, 2022) are reported, but lack the validation of CEP function by genetic studies. Further work, including in other legume species such as Lotus, is required to get a better understanding of if and how strongly the identified regulatory mechanisms in Medicago are conserved across legumes.

### Negative regulation of SlCEP2–SlCEPR1 signalling correlates with promotion of the AM symbiosis with tomato

In addition to legume–rhizobial symbioses, recent evidence suggests that suppression of CEP–CEPR1 signalling is required in tomato to promote AM symbiosis. Hsieh *et al.* (2022) provided genetic and transcriptional analyses suggesting that the establishment of AM symbiosis down-regulates *SlCEP2,* but not other CEP genes, to increase LR number via enhancement of auxin biosynthesis and transport in an *SlCEPR1-*dependent manner. The ultimate impact of *SlCEP2* suppression on the progression of the AM symbiosis (Fusconi, 2014) and on plant nutrient acquisition, however, requires further investigation.

## Immunity

Recently, work by Fitrianti *et al.* (2022) using addition of an AtCEP5 variant suggested a potential role for CEPs in Arabidopsis defence responses to non-adapted fungal and bacterial pathogens, possibly via a mechanism independent of CEPR1 and CEPR2. Work by Rzemieniewski *et al.* (2022, Preprint) has provided evidence clarifying the roles of CEPs and CEP receptors in resistance responses to plant pathogenic bacteria in Arabidopsis. The authors used genetics, biochemical approaches, grafting, and *in vitro* and *in vivo* plant immunology assays to show that CEPs play a role in triggering several typical immune outputs and that this involved CEP expression and perception in the shoot by CEPR1, CEPR2, and RECEPTOR-LIKE KINASE 7 (RLK7). RLK7 was previously shown to perceive CEP-related endogenous PAMP-INDUCED PEPTIDES (PIPs) (Hou *et al.*, 2014). CEP4, which has an unusual structure relative to other CEP family members, specifically interacted with CEPR2 and RLK7. Rzemieniewski *et al.* (2022, Preprint) provided evidence that the increased resistance to pathogens observed under low N was mediated by CEP induction under these conditions. They confirmed that low N enhances the flagellin 22 (flg22)-triggered activation of MITOGEN-ACTIVATED PROTEIN KINASEs (MAPKs) and *FLAGELLIN-INDUCED RECEPTOR KINASE 1* (*FRK1*), and showed that this response is abolished in a CRISPR/Cas9-derived knockout mutant in six of the 12 Class 1 CEP genes. The results suggest that CEPs play a role in coordinating immune responses with growth and environmental cues and may point to CEPs being important players in the trade-off between growth, development, and immunity (Rzemieniewski *et al.*, 2022, Preprint).

## Nematode infection

CEP-coding genes were found outside seed plants in the genomes of root-parasitic nematodes, but not other nematodes (Bobay *et al.*, 2013; Delay *et al.*, 2013; Eves-Van Den Akker *et al.*, 2016; Mishra *et al.*, 2023). Sedentary root-knot nematodes (*Meloidogyne* spp.) and *Rotylenchulus reniformis* spp. encoded between seven and 16 CEP mimic genes, suggesting an adaptive advantage of CEP genes for root nematodes. The structure of these gene mimics is distinct from those of seed plants, thus it is unlikely that nematodes acquired them through horizontal gene transfer. CEP mimics may have roles in increasing host N uptake and regulating the size of the nematode feeding site (Eves-Van Den Akker *et al.*, 2016; Mishra *et al.*, 2023), which is akin to root nodules, as both are sink tissues that require a flow of nutrients to support the initiation of organ growth. Given plant CEP involvement in root lateral organogenesis and that root-parasitic nematodes penetrate and trigger feeding site formation in the zone of elongation (Goverse *et al.*, 2000; Caillaud *et al.*, 2008), it seems plausible that nematode CEPs mimic plant CEPs to enable the formation of feeding sites. Plant-parasitic nematodes use their stylets to deliver secretions into the host plant tissue or cells. These secretions include effector proteins that suppress host defences and manipulate plant development (Hewezi and Baum, 2013). Since CEPRs are predominantly located on the plant plasma membrane, it is possible that nematode CEP mimics could bind to these receptors and activate host CEP signalling pathways. It is plausible that nematode CEP mimics manipulate plant development and nutrient demand to create a favourable environment for the nematode to feed and reproduce, but further work is required to confirm this.

## Conclusions

In conclusion, CEP signalling via CEPRs is a conserved pathway in flowering plants that plays crucial roles in regulating a wide range of processes including nutrient uptake, root system architecture, reproductive development, and interactions with plant microbes and parasites. CEPs act as long-distance signals to integrate external nutrient availability with internal nutrient demand, facilitating compensatory nutrient uptake in roots exposed to high nutrient levels. CEPs also regulate LR and nodule development, with the specificity of different CEP ligands influencing the balance between these two processes. The conservation of CEP signalling across plant species suggests that it may be a promising target for improving crop productivity by improving nutrient uptake and usage. In legumes, promoting CEP signalling could enhance nodulation, leading to improved N fixation and increased crop yields. CEPs could also be used to pause root growth under nutrient limitation, allowing plants to conserve energy while still scavenging for critical nutrients. Understanding the molecular mechanisms underlying CEP–CEPR signalling could provide new avenues for improving plant nutrient acquisition and adaptation to nutrient limitation, potentially leading to more sustainable agriculture practices.
